# RDE: A novel approach to improve the classification performance and expressivity of KDB

**DOI:** 10.1371/journal.pone.0199822

**Published:** 2018-07-23

**Authors:** Hua Lou, LiMin Wang, DingBo Duan, Cheng Yang, Musa Mammadov

**Affiliations:** 1 Changzhou College of Information Technology, ChangZhou, China; 2 College of Computer Science and Technology, Jilin University, ChangChun, China; 3 Faculty of Science and Technology, Federation University, Ballarat, Australia; Universitatsmedizin Greifswald, GERMANY

## Abstract

Bayesian network classifiers (BNCs) have demonstrated competitive classification performance in a variety of real-world applications. A highly scalable BNC with high expressivity is extremely desirable. This paper proposes Redundant Dependence Elimination (RDE) for improving the classification performance and expressivity of *k*-dependence Bayesian classifier (KDB). To demonstrate the unique characteristics of each case, RDE identifies redundant conditional dependencies and then substitute/remove them. The learned personalized *k*-dependence Bayesian Classifier (PKDB) can achieve high-confidence conditional probabilities, and graphically interpret the dependency relationships between attributes. Two thyroid cancer datasets and four other cancer datasets from the UCI machine learning repository are selected for our experimental study. The experimental results prove the effectiveness of the proposed algorithm in terms of zero-one loss, bias, variance and AUC.

## Introduction

Data mining is the analysis step of the “knowledge discovery in databases” process and its goal is the extraction of patterns and knowledge from large amounts of data. During the past decades, statistical models, such as Bayesian network, neural network and support vector machine, have been proposed and applied in many real life applications, e.g. precision medicine. Due to the high prediction performance of these statistical models, researchers would like to gain an understanding of the reasons behind such a prediction, especially when the prediction contradicts their intuition. For example, physicians are typically not only interested in the final prediction, but also like to understand the underlying inference procedure that may help explain why the system makes a certain recommendation. An explanatory, causal and graphical model is more desirable to visualize and mine previously undiscovered knowledge from data [1].

Bayesian network classifiers (BNCs) have long been a popular tool for graphically representing the probabilistic dependencies and inferring under conditions of uncertainty [2–5]. Numerous BNCs (e.g., Naive Bayes (NB) [6], tree augmented Naive Bayes (TAN) [7], Averaged One-Dependence Estimators (AODE) [8] and *k*-dependence Bayesian classifier (KDB) [9–11] have been proposed to mine dependency relationships from data. Among them, KDB can generalize to describe any higher degrees of attribute dependence. KDB provides the “average network” to express significant dependencies and this “one size fits all” solution obviously cannot apply to all cases. Patients with similar symptoms may have different kinds of diseases. For example, because of low incidence rate, AIDS (Acquired Immune Deficiency Syndrome) at early stage is often diagnosed as influenza [12]. How to enable person to have “personalized network”, which can describe the dependency relationships among specific characteristics or attributes for each case, is still challenging. Local graph structure ${\text{KDB}}_{P}$ [2] takes each case or unlabeled testing instance P as a target and can describe local causal relationships implicated. However, the number of conditional dependencies in ${\text{KDB}}_{P}$ is determined by user-specified parameter *k*. Some redundant dependencies should be replaced with more meaningful or “personalized” dependencies that only hold in specific instances.

In this paper, a new approach, called Redundant Dependency Elimination (RDE), is proposed to identify redundant conditional dependencies in ${\text{KDB}}_{P}$ and then substitute/remove them at classification time. The resulting optimized network structure of ${\text{KDB}}_{P}$, denoted by KDB_*O*_, can increase the confidence level of conditional probabilities. The final personalized classifier, PKDB, is an ensemble of KDBs learned from training data and testing instance respectively. PKDB combines the computational efficiency of classical generative learning with the control of bias/variance trade-off. Two thyroid disease datasets and four other cancer datasets from the UCI machine learning repository are selected for our experimental study. The experimental results show the advantages of PKDB over other classifiers.

## Materials and methods

### Classifiers

LibSVM [13] and Random forest [14] are introduced in this paper for comparison study. We use Weka’s implementations and default settings of Random forest with the exceptions of 20 decision trees. We use Weka’s implementations and default settings of LibSVM and performing a “grid-search” on *C* and *γ* for the RBF kernel using 5-fold cross-validation. Each pair of (*C*, *γ*) is tried (*C* = 2^−5^, 2^−3^, ⋯, 2^15^, *γ* = 2^−15^, 2^−13^, ⋯, 2^3^) and the one with the lowest cross-validation zero-one loss is selected. For clarity, the abbreviation of algorithms mentioned above is shown in Table 1.

**Table 1:** Abbreviation of algorithms introduced in this paper.

Index	Description	Abbreviation
1	Redundant dependency elimination	RDE
2	KDB learned from training data	KDB
3	KDB learned from testing instance P	${\text{KDB}}_{P}$
4	Ensemble of KDB and ${\text{KDB}}_{P}$	AKDB
5	${\text{KDB}}_{P}$ optimized by RDE	KDB_*O*_
6	Ensemble of KDB and KDB_*O*_	PKDB
7	A Library for Support Vector Machines	LibSVM
8	Random forest	RF

### Data

Six datasets from UCI machine learning repository [15] are selected in this paper for case study. The detailed introduction of these datasets are shown in Table 2, which summarizes the characteristics of each dataset, including the numbers of instances, attributes and classes. For each benchmark dataset, we use MDL discretization [16] to discretize quantitative attributes using 3-bin equal frequency discretization.

**Table 2:** Description of data sets.

Index	Data set	Case	Att	Class
1	Dis	3772	29	2
2	Hypothyroid	3163	25	2
3	Breast-cancer-w	699	9	2
4	Haberman	306	3	2
5	Heart-disease-c	303	13	2
6	Pima-ind-diabetes	768	8	2

### Metrics

Zero-one loss is one of the most commonly used metrics to measure the classification performance of a classifier. Zero-one loss can measure how well a classifier correctly identifies or discriminate an unlabeled instance. Let **X** and *Y* be the input and output spaces respectively, and elements **x** and *y* respectively. The zero-one loss function for instance **x** is defined as [17]:

$$\begin{array}{c} \xi \left(x\right)=1-\delta \left(y,\hat{y}\right), \end{array}$$

where $\delta (y,\hat{y})=1$ if $\hat{y}=y$ and zero otherwise, *y* and $\hat{y}$ are respectively the true class label and predicted label of **x**. Kohavi and Wolpert presented a bias-variance decomposition of the zero-one loss function [17]. The bias term measures the squared difference between the average output of the target and the algorithm, and it is defined as follows [17]:

$$\begin{array}{c} bias\left(x\right)=\frac{1}{2}\sum_{y^{\prime }\epsilon Y}{\left[P\left(y^{\prime }|x\right)-P\left(y|x\right)\right]}^{2}, \end{array}$$

The variance term measures the sensitivity of the algorithm to the changes in the training set, and it is defined as follows [17]:

$$\begin{array}{c} variance\left(x\right)=\frac{1}{2}\left[1-\sum_{y^{\prime }\epsilon Y}P{\left(y^{\prime }|x\right)}^{2}\right]. \end{array}$$

In machine learning, the bias-variance tradeoff is a central problem for supervised learning. Ideally, one wants to choose a model that both accurately captures the regularities in its training data, but also generalizes well to unseen data. Unfortunately, it is typically impossible to do both simultaneously. High-variance learning methods (e.g., high-dependence BNCs) are usually more complex, enabling them to capture more complex multivariate relationships, but at risk of overfitting to noisy or unrepresentative training data. In contrast, high-bias component of zero-one loss is highly appealing to simpler models that don’t tend to overfit, but may underfit their training data, failing to capture important regularities.

The statistical hypothesis test, e.g. Friedman test [18], can test the null hypothesis of no differences between algorithms. Friedman test ranks the algorithms for each data set separately: the best performing algorithm getting the rank of 1, the second best ranking 2, and so on. In case of ties, average ranks are assigned. The Friedman statistic can be computed as follows [18]:

$$\begin{array}{c} X_{F}^{2}=\frac{12}{Nt(t+1)}\sum_{j=1}^{n}R_{j}^{2}-3N\left(t+1\right), \end{array}$$ (1)

where $R_{j}=\sum_{i}r_{i}^{j}$ and $r_{i}^{j}$ is the rank of the *j*-th of *t* algorithms on the *i*-th of *N* datasets.

Sensitivity measures the proportion of actual positives that are correctly identified and specificity measures the proportion of actual negatives that are correctly identified. Receiver operating characteristic curve, i.e. ROC curve, is a powerful tool to illustrate the diagnostic ability of a binary classifier by plotting the true positive rate (Sensitivity) against the false positive rate (100-Specificity) for different cut-off points. Each point on the ROC curve represents a sensitivity/specificity pair corresponding to a particular decision threshold. The ROC curve graphically displays the trade-off between sensitivity and specificity and is useful in assigning the best cut-offs. The area under the ROC curve (AUC) [19] provides a simple numeric measure indicating the performance over the visual comparison of ROC curves.

## Bayesian network classifiers

Given class variable *Y* and a set of discrete attributes **X** = {*X*_1_, *X*_2_, ⋯, *X*_*n*_} (In the following formulas, all variables are assumed to be discrete.) the aim of supervised learning is to predict the discrete class label *y* of a testing instance **x** = (*x*_1_, ⋯, *x*_*n*_), where *x*_*i*_ is the value of attribute *X*_*i*_ and *y* is the value of class variable *Y*. The restricted BNCs, e.g., KDB, model joint probability distribution *P*(**x**, *y*) according to chain rule, which can be described in the form of a product of a set of conditional probabilities.

$$\begin{array}{c} P\left(x,y\right)=P\left(y\right)\prod_{i=1}^{n}P\left(x_{i}|\Pi_{i},y\right), \end{array}$$ (2)

where Π_*i*_ represents the parent attribute set of *X*_*i*_.

From the definition of conditional probability, we use the following Formula to classify

$$\begin{array}{c} P\left(y|x\right)=\frac{P(x,y)}{P(x)}=\frac{P(x,y)}{\sum_{y}P\left(x,y\right)}. \end{array}$$ (3)

When attribute number *n* is high and/or data size *N* is relatively small, it would be difficult to obtain a sufficiently accurate estimate of *P*(*x*_*i*_|Π_*i*_, *y*) from the sample frequencies. One popular solution is to restrict the number of parents of each attribute while trying to retain accurate estimate of *P*(*x*_*i*_|Π_*i*_, *y*). That is, given attribute subset ${\hat{\Pi }}_{i}\subset \Pi_{i}$, $P\left(x_{i}|{\hat{\Pi }}_{i},y\right)\approx P\left(x_{i}|\Pi_{i},y\right)$ holds. Sahami [11] proposed the notion of *k*-dependence BNC, which allows each attribute *X*_*i*_ to have a maximum of *k* attribute nodes as parents.

NB is the simplest of the BNCs, assuming that all attributes are independent given the class. There exist no dependency relationships between attributes and thus NB is a 0-dependence BNC. AODE utilizes a restricted class of one-dependence estimators (ODEs) and aggregates the predictions of all qualified estimators within this class. TAN relaxes NB’s independence assumption by allowing every attribute to have at most one other attribute as parent. Its basic structure extends the Chow-Liu tree [20] to a maximum spanning tree. The arc or conditional dependence between attributes *X*_*i*_ and *X*_*j*_ is measured by conditional mutual information (CMI) *I*(*X*_*i*_; *X*_*j*_|*Y*) given class variable, which is defined as follows [21],

$$\begin{array}{c} I\left(X_{i};X_{j}|Y\right)=\sum_{x_{i}}\sum_{x_{j}}\sum_{y}P\left(x_{i},x_{j},y\right)log\frac{P(x_{i},x_{j}|y)}{P\left(x_{i}|y\right)P\left(x_{j}|y\right)} \end{array}$$ (4)

KDB further relaxes NB’s independence assumption by allowing any attribute *X*_*i*_ to be conditioned on at most *k* other attributes, i.e., at most *k* arcs from other attributes to *X*_*i*_. Unlike TAN, KDB requires to determine the attribute order by comparing the mutual information (MI) *I*(*X*_*i*_; *Y*) between attribute *X*_*i*_ and class *Y*, which is defined as follows [21],

$$\begin{array}{c} I\left(X_{i};Y\right)=\sum_{x_{i}}\sum_{y}P\left(x_{i},y\right)log\frac{P(x_{i},y)}{P\left(x_{i}\right)P\left(y\right)} \end{array}$$ (5)

The learning procedures of KDB is described in Algorithm 1.

**Algorithm 1**: Structure learning of KDB

**Input:** Training set T, parameter *k* = 2, crosstab *CMI* = {*I*(*X*_*i*_, *X*_*j*_|*Y*)|1 ≤ *i* ≠ *j* ≤ *n*} (see formula (4)) and vector *MI* = {*I*(*X*_*i*_; *Y*)|1 ≤ *i* ≤ *n*} (see formula (5)).

**Output:** Network structure ${\text{KDB}}_{T}=\left\{V,E\right\}$, where V is the node set and E is the edge set.

1 Let L be a list of all *X*_*i*_ in descending order of *I*(*X*_*i*_; *Y*).

2 V={Y}; E=⌀;

3 **for** *i* = 1 → *n* **do**

4  V=V∪L[i];

5  E=E∪(Y→L[i]);

6 **end**

7 **for** *i* = 1 → *n* **do**

8  S=⌀;

9  $\hat{k}=k$;

10  **while** ($\hat{k}>0$) **do**

11   $m=\arg\max_{j}\left\{I\left(L\left[i\right];L\left[j\right]|Y\right):1\leq j<i\right),j\notin S\}$;

12   E=E∪(L[m]→L[i]);

13   $\hat{k}=\hat{k}-1$;

14   *S* = *S* ∪ {*m*};

15  **end**

16 **end**

17 **return** ${\text{KDB}}_{T}$

**Algorithm 2**: Structure learning of ${\text{KDB}}_{P}$

**Input:** testing instance P, parameter *k* = 2, vector LMI = {*I*(*x*_*i*_;*Y*)|1 ≤ *i* ≤ *n*}, crosstab CLMI = {*I*(*x*_*i*_, *x*_*j*_|*Y*)|1 ≤ *i* ≠ *j* ≤ *n*} (see formula (6)).

**Output:** Network structure ${\text{KDB}}_{P}=\left\{V,E\right\}$, where V is the node set and E is the edge set.

1 Let L be a list of all *x*_*i*_ in descending order of *I*(*x*_*i*_;*Y*).

2 V={Y}; E=⌀;

3 **for** *i* = 1 → *n* **do**

4  V=V∪L[i];

5  E=E∪(Y→L[i]);

6 **end**

7 **for** *i* = 1 → *n* **do**

8  S=⌀;

9  $\hat{k}=k$;

10  **while** ($\hat{k}>0$) **do**

11   $m=\arg\max_{j}\left\{I\left(L\left[i\right];L\left[j\right]|Y\right):1\leq j<i\right),j\notin S\}$;

12   E=E∪(L[m]→L[i]);

13   $\hat{k}=\hat{k}-1$;

14   *S* = *S* ∪ {*m*};

15  **end**

16 **end**

17 **return** ${\text{KDB}}_{P}$

KDB can represent the “average knowledge” or “expert knowledge” mined from data, that roughly describes the dependency relationships between different inputs, e.g., the dependency relationship between {*Gender*, *Age*} and *TSH*. However, KDB cannot finely describe the dependency relationships in different patient records, e.g., the relative independency relationship between {*Gender* = “*male*”, *Age* = 20} and *TSH* = “*yes*”, or the relative dependency relationship between {*Gender* = “*female*”, *Age* = 45} and *TSH* = “*yes*”. In contrast, ${\text{KDB}}_{P}$ represents “personalized knowledge” mined from instance P. The “average knowledge” learned from labeled training data and the “personalized knowledge” learned from unlabeled testing instance are complementary in nature. Thus they should be considered simultaneously for classification. To achieve this goal, ${\text{KDB}}_{P}$ applies the same learning strategy that KDB uses. Given testing instance $P=(x_{1},\cdots ,x_{n})$, ${\text{KDB}}_{P}$ sorts attributes by comparing local mutual information (LMI) *I*(*x*_*i*_; *x*_*j*_|*Y*) and choose appropriate conditional dependencies by comparing conditional local mutual information (CLMI). LMI and CLMI are defined as follows [2],

$$\begin{array}{c} \left\{ \begin{array}{cc} & I\left(x_{i};Y\right)=\sum_{y}P\left(x_{i},y\right)\log\frac{P(x_{i},y)}{P\left(y\right)P\left(x_{i}\right)}\\ & I\left(x_{i};x_{j}|Y\right)=\sum_{y}P\left(x_{i},x_{j},y\right)\log\frac{P(x_{i},x_{j}|y)}{P\left(x_{i}|y\right)P\left(x_{j}|y\right)} \end{array}\right. \end{array}$$ (6)

From the viewpoint of information theory, MI or *I*(*X*_*i*_; *Y*) can measure the uncertainty reduction in *Y* given the information from *X*_*i*_. The attributes corresponding to greater reduction will get higher rank and added to the network structure in priority. By comparing formulas (5) and (6) we can see that, *I*(*X*_*i*_; *Y*) = ∑_*x*_*i*__ *I*(*x*_*i*_; *Y*). MI refers to the average of all possible events, and it is the expected value of LMI over all possible values of *X*_*i*_. LMI can be used to measure the uncertainty reduction in *Y* given the information from *X*_*i*_ = *x*_*i*_. Because *I*(*X*_*i*_; *X*_*j*_|*Y*) = ∑_*x*_*i*_, *x*_*j*__ *I*(*x*_*i*_; *x*_*j*_|*Y*), we can get similar results that *I*(*x*_*i*_; *x*_*j*_|*Y*) can measure the conditional dependence between *X*_*i*_ and *X*_*j*_ when they take specific values.

The ensemble of KDB and ${\text{KDB}}_{P}$, i.e., AKDB [2], has better overall prediction accuracy, on average, than any individual member. KDB and ${\text{KDB}}_{P}$ apply the same learning strategy whereas model different data spaces (training data and testing instance). It is difficult to judge which output from these two classifiers should be considered in priority. The linear combiner is used for models that output real-valued numbers, so is applicable for BNC. In practice, it is inappropriate to pre-determine the weight of subclassifier. Thus in practice AKDB uses the uniformly rather than nonuniformly weighted average. The ensemble probability estimate is

$$\begin{array}{c} \hat{P}\left(y|x,\text{AKDB}\right)=\frac{P\left(y|x,\text{KDB}\right)+P\left(y|x,{\text{KDB}}_{P}\right)}{2}. \end{array}$$ (7)

Given *m* class labels, the class label y* of unlabeled instance **x** corresponds to the highest value of posterior probability of $\hat{P}\left(y|x,\text{AKDB}\right)$, where *y* ∈ {*y*_1_, ⋯, *y*_*m*_}, i.e.,

$$\begin{array}{c} y^{*}=\arg\max\hat{P}\left(y|x,\text{AKDB}\right). \end{array}$$ (8)

The classification procedure of AKDB is shown in Algorithm 3.

**Algorithm 3**: Classification procedure of AKDB

**Input:** testing instance $P=(x_{1},\cdots ,x_{n})$, KDB learned from Algorithm 1 and ${\text{KDB}}_{P}$ learned from Algorithm 2.

**Output:** Class label y*.

1 Compute the joint probability *P*(*y*,**x**|KDB) and $P(y,x|{\text{KDB}}_{P})$ by Formula (2);

2 Compute the conditional probability *P*(*y*|**x**, KDB) and $P(y|x,{\text{KDB}}_{P})$ by Formula (3);

3 Compute the conditional probability *P*(*y*|**x**, AKDB) by Formula (7);

4 Compare and predict the class label y* for P by Formula (8);

5 **Return** y*;

## Redundant dependency elimination

Suppose that Π_*i*_ = {*X*_1_, ⋯, *X*_*i*−1_}, from the chain rule of mutual information we have [21]

$$\begin{array}{ccc} I(X_{i};\Pi_{i},Y) & = & I\left(X_{i};Y\right)+I\left(X_{i};X_{1}|Y\right)+I\left(X_{i};X_{2}|X_{1},Y\right)+\\ & & \cdots +I(X_{i};X_{i-1}|X_{1},\cdots ,X_{i-2},Y) \end{array}$$ (9)

KDB implicitly reduces *I*(*X*_*i*_; *X*_*j*_|*X*_1_, ⋯, *X*_*j*−1_, *Y*) to *I*(*X*_*i*_; *X*_*j*_|*Y*) when *j* > 1. The same strategy is also applicable to ${\text{KDB}}_{P}$. Obviously, the dependency relationships between the parent attributes of *X*_*i*_ are neglected, that will inevitably result in estimation bias. For different instances, the dependency relationships may differ. Here, we introduce Pointwise mutual information (PMI) *I*(*x*_*i*_; *x*_*j*_) and Pointwise conditional mutual information (PCMI) *I*(*x*_*i*_; *x*_*k*_|*x*_*j*_) to address this issue. The definitions of PMI and PCMI are as follows [22],

$$\begin{array}{c} \left\{ \begin{array}{cc} & I\left(x_{i};x_{j}\right)=log\frac{P(x_{i},x_{j})}{P\left(x_{i}\right)P\left(x_{j}\right)}\\ & I\left(x_{i};x_{k}|x_{j}\right)=log\frac{P(x_{i},x_{k}|x_{j})}{P\left(x_{i}|x_{j}\right)P\left(x_{k}|x_{j}\right)} \end{array}\right. \end{array}$$ (10)

The dependency relationships in ${\text{KDB}}_{P}$ that are relevant or irrelevant to class labels are respectively measured by formulas (6) and (10), the confidence levels of which are determined by the estimation of probability distributions. The probability distributions have to be estimated from training data before structure learning. For small datasets, the sparsely distributed attribute values make the estimation of lower-order probability estimations much more reliable than that of the higher-order ones. If the probability distributions learned from training data are not reliable, the resulting non-robust classifier will make wrong prediction. That may be the main reason why NB offers competitive performance with high efficiency, strong robustness and loose coupling on some small datasets.

PMI and PCMI refer to single events. Like MI, PMI also follows the chain rule, i.e.,

$$\begin{array}{c} I\left(x_{i};x_{1},\cdots ,x_{i-1}\right)=I\left(x_{i};x_{1}\right)+I\left(x_{i};x_{2}|x_{1}\right)+\cdots +I\left(x_{i};x_{i-1}|x_{1},\cdots ,x_{i-2}\right) \end{array}$$ (11)

**Fig 1:**
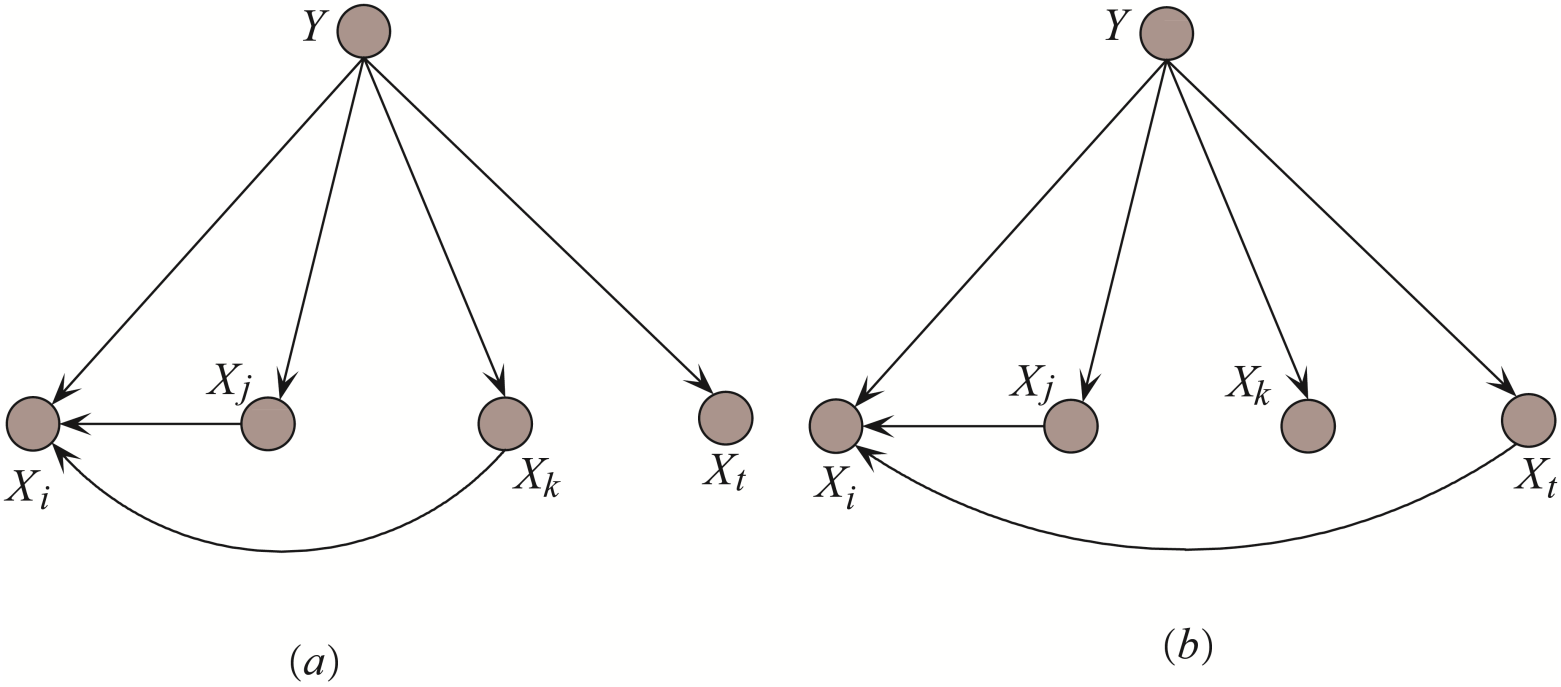
Example: Conditional dependencies between *X*_*i*_ and its parents. (a) *X*_*i*_ has two parent attributes *X*_*j*_ and *X*_*k*_. (b) Parent attribute *X*_*k*_ is substituted with *X*_*t*_.

In computational linguistics, PMI has been used for finding co-occurrences of words in a text corpus and to approximate the probabilities *P*(*x*) and *P*(*x*, *y*) respectively. MI can roughly measure the dependency relationship between the associated variables, but cannot measure the inherent relational mapping between specific variable values. Given two attributes *X*_*i*_, *X*_*j*_, each having two values and <*X*_*i*_, *X*_*j*_ > = {(1, 1), (1, 2), (2, 2)} for example, obviously when *X*_*i*_ = 1 the uncertainty of *X*_*j*_ reaches a maximum, whereas when *X*_*i*_ = 2 the uncertainty of *X*_*j*_ is reduced to zero. In practice, it may be the case that certain values are more significant than others, or that certain patterns of association are more semantically important than others. Further, it is desirable to obtain reasonable causal relationships for causality analysis rather than a simple classification result. Considering an example of a substructure shown in Fig 1(a). Corresponding training data is presented in Table 3. In this example, *X*_*i*_ has two parents *X*_*j*_, *X*_*k*_ and its conditional probability is *P*(*x*_*i*_|*x*_*j*_, *x*_*k*_, *y*).

**Table 3:** An example of training data with four attributes, in which the mapping relationships between {*X*_*i*_, *X*_*j*_, *X*_*k*_} are shown.

*X*_*i*_	*X*_*j*_	*X*_*k*_	*X*_*t*_
*a*	*c*	*e*	*b*
*b*	*d*	*e*	*b*
*b*	*d*	*e*	*c*
*b*	*d*	*e*	*c*
*a*	*d*	*e*	*c*
*a*	*c*	*f*	*d*

KDB or ${\text{KDB}}_{P}$ just consider the conditional dependence between *X*_*i*_ and its parents, and the relationships among parents are neglected, that may not help to increase the confidence level of *P*(*x*_*i*_|Π_*i*_, *y*) or reduce the uncertainty of *X*_*i*_ when it takes specific values. For testing instance P, its class label is unknown thus Redundant Dependency Elimination (RDE) just considers the dependency relationships between attribute values. For example, given {*X*_*i*_ = *b*, *X*_*j*_ = *d*, *X*_*k*_ = *e*} in Table 3, from the chain rule of PMI we will have

$$\begin{array}{ccc} I(x_{i};x_{j},x_{k}) & = & I\left(x_{i};x_{j}\right)+I\left(x_{i};x_{k}|x_{j}\right)=\log\frac{P(b,d)}{P(b)P(d)}+\log\frac{P(b,e|d)}{P(b|d)P(e|d)}\\ & = & \log\frac{P(b,d)}{P(b)P(d)}+\log\frac{P(b,d,e)P(d)}{P(b,d)P(d,e)}\\ & = & \log\frac{\frac{1}{2}}{\frac{1}{2}\cdot \frac{4}{6}}+\log\frac{\frac{1}{2}\cdot \frac{4}{6}}{\frac{1}{2}\cdot \frac{4}{6}}\\ & = & \log\frac{6}{4}+0=\log\frac{6}{4} \end{array}$$ (12)

Thus *I*(*x*_*i*_; *x*_*j*_, *x*_*k*_) = *I*(*x*_*i*_; *x*_*j*_), i.e., *x*_*k*_ does not provide any extra valuable information to reduce the uncertainty of *x*_*i*_. To further increase the conditional probability of *x*_*i*_, we should select another attribute value, e.g., *x*_*t*_, to take the place of *x*_*k*_. If *I*(*x*_*i*_; *x*_*t*_|*x*_*j*_) > 0, then

$$\begin{array}{c} I\left(x_{i};x_{j},x_{t}\right)-I\left(x_{i};x_{j},x_{k}\right)=\left[I\left(x_{i};x_{j}\right)+I\left(x_{i};x_{t}|x_{j}\right)\right]-I\left(x_{i};x_{j}\right)=I\left(x_{i};x_{t}|x_{j}\right)>0 \end{array}$$ (13)

Thus the larger the difference is, the more appropriate *X*_*t*_ is as the parent of *X*_*i*_. If the attributes are sorted by comparing *I*(*x*_*i*_; *Y*) and the resulting order is {*x*_1_, ⋯, *x*_*n*_}, then *x*_*i*_ can select at most *k* parents from *i* − 1 attributes that ranks higher. Suppose that its parents are sorted by comparing *I*(*x*_*i*_; *x*_*j*_|*Y*)(*j* < *i*) and the order is $\left\{{\hat{x}}_{1},\cdots ,{\hat{x}}_{i-1}\right\}$, RDE first operates by iteratively identifying redundant parents of each attribute. It uses the criterion

$$\begin{array}{c} \frac{I(x_{i};{\hat{x}}_{j}|{\hat{x}}_{1})}{I(x_{i};{\hat{x}}_{1})}\geq \delta \end{array}$$ (14)

to infer that except the information ${\hat{x}}_{1}$ provides to *x*_*i*_, ${\hat{x}}_{j}$ can provide extra information to *x*_*i*_, where ${\hat{X}}_{j}\in \Pi_{i}$ and 1 < *j* ≤ *i* − 1, *δ* is a minimum redundancy ratio. If there exist attribute values that make formula (14) hold, then an appropriate parent of *x*_*i*_ should be selected from them. This process is terminated if there is no redundancy or no substituted attribute available. We keep the attribute value with the smallest index and disregard the other attribute values. For instance, if ${\hat{x}}_{2},{\hat{x}}_{3}$ and ${\hat{x}}_{4}$ hold for formula (14), we only take ${\hat{x}}_{2}$ as the parent of *x*_*i*_.

Starting from the basic network structure learned from testing instance, KDB_*O*_ repairs “harmful” interdependencies by applying RDE to remove highly correlated attribute values in classification time. Note that attribute selection approaches, such as Backwards sequential elimination (BSE, [23, 24]), simply remove attributes to achieve zero-one loss improvement. BSE operates by iteratively removing successive attributes until no zero-one loss improvement. According to Formula 2, attribute *X*_*i*_ can have at most *i* − 1 parents, i.e., there exists *i* − 1 conditional dependencies between *X*_*i*_ and its parents. If *X*_*i*_ is removed from Bayesian network structure, then *i* − 1 conditional dependencies will be implicitly removed correspondingly. That will result in great change in network structure and classification bias. In contrast, RDE retains all attributes and resolve such interdependencies with much more flexible strategy and finer tuning, as for some test instances one conditional dependence may be identified as redundant and then substituted or removed, for other test instances it may hold.

One effective way of resolving the trade-off between bias and variance is to use ensemble learning [9, 25]. For example, boosting combines many “weak” (high bias) models in an ensemble that has lower bias than the individual models, while bagging combines “strong” learners in a way that reduces their variance. KDB and KDB_*O*_ are both “strong” learners. KDB takes training set as a target and build general BNC for it. KDB_*O*_ takes testing instance P as a target and build a specific BNC for P. In contrast to KDB, KDB_*O*_ is defined by the conditional dependencies at the attribute values in P. Obviously, for different testing instances, KDB remains the same while KDB_*O*_ may differ greatly. RDE identifies and then substitutes/removes the redundant dependencies in ${\text{KDB}}_{P}$, that will make the conditional dependencies in KDB_*O*_ much more reasonable.

The final model, PKDB, is an ensemble of KDB and KDB_*O*_. The ensemble probability estimate for PKDB is

$$\begin{array}{c} \hat{P}\left(y|x,\text{PKDB}\right)=\frac{P\left(y|x,\text{KDB}\right)+P\left(y|x,{\text{KDB}}_{O}\right)}{2}. \end{array}$$

PKDB can represent arbitrary *k*-dependence relationships. It seems that PKDB with higher degree of attribute dependence will more closely fit the training data and can achieve better generalization performance than those with lower degree of attribute dependence. However, higher degree of attribute dependence needs more training instances to ensure more accurate estimation of conditional probability. From Table 2, the thyroid disease datasets for experimental study contain relatively small number (< 3800) of instances but large number (≥ 25) of attributes. To make resulting algorithm combine the computational efficiency of classical generative learning with the control of bias/variance trade-off, in the following discussion we restrict PKDB to be 2-dependence, i.e., *k* = 2, as used in [2]. Since attribute *X*_*i*_ can have *k* parent attributes with higher ranks, the problem of redundant dependency arises when *i* ≥ *k* + 2. The detailed learning procedure of PKDB is presented in Algorithm 4.

**Algorithm 4**: Redundant Dependency Elimination for KDB_*O*_ when *k* = 2

**Input:** Network structure ${\text{KDB}}_{P}$, parameter *k*, testing instance P.

**Output:** KDB_*O*_, network structure after applying RDE.

1 Transform ${\text{KDB}}_{P}$ to a set of children-parent pairs {*x*_1_, Π_1_} ⋯, {*x*_*n*_, Π_*n*_}.

2 Let L be a list of all *x*_*i*_ in descending order of *I*(*x*_*i*_; *Y*).

3 **for** *i* = *k* + 2 → *n* **do**

4  Let $L^{\prime }$ be a list of all *x*_*j*_(*x*_*j*_ ∈ Π_*i*_) in descending order of *I*(*x*_*i*_; *x*_*j*_|*Y*);

5  $\Pi_{i}=\left\{L^{\prime }\left[1\right]\right\}$;

6  **for** *j* = 2 → *i* − 1 **do**

7   **if** ($I\left(L\left[i\right];L^{\prime }\left[j\right]|L^{\prime }\left[1\right]\right)\geq \delta \cdot I\left(L\left[i\right];L^{\prime }\left[1\right]\right)$) (see formula (14)) **then**

8    $\Pi_{i}=\left\{L^{\prime }\left[1\right],L^{\prime }\left[j\right]\right\}$;

9   **end**

10  **end**

11 **end**

12 Transform revised children-parent pairs {*x*_1_, Π_1_} ⋯, {*x*_*n*_, Π_*n*_} to KDB_*O*_.

13 **return** KDB_*O*_

During training PKDB generates a three-dimensional table of co-occurrence counts for each pair of attribute values and each class value to estimate the probabilities *P*(*y*), *P*(*x*_*i*_, *y*), *P*(*x*_*i*_, *x*_*j*_, *y*), *P*(*x*_*i*_, *x*_*j*_) and *P*(*x*_*i*_, *x*_*j*_, *x*_*k*_). KDB requires *O*(*Nm*(*nv*)^2^) time (dominated by calculating CMI) [11] to build the network structure, where *v* is the average number of discrete values that an attribute may take. The basic structure of KDB_*O*_ only considers the attribute values in testing instance and thus requires *O*(*Nmn*^2^) time. RDE requires *O*(*Nn*^2^) time to calculate PCMI, then an extra pass is needed to perform identification and then substitute/remove redundant conditional dependencies. The final time complexity for building KDB_*O*_ is *O*(*Nmn*^2^) + *O*(*Nn*^3^). The time complexities of classifying a single instance for KDB and KDB_*O*_ are the same, *O*(*mnk*).

## Results

The experimental system is implemented in C++. The experiments are conducted on a desktop computer with an Intel(R) Core(TM) i5-7200 CPU @3.20GHz, 64 bits and 12,288 MB of memory. For the BNCs to be compared, 10-fold cross validation is applied to obtain an accurate estimation of the average performance. For each fold, leave-one-out cross validation zero-one loss [26] [27] is used as selection criterion to determine *δ* in Formula (14). Table 4 summarizes the experimental results in terms of zero-one loss, bias, variance and AUC. The Friedman statistic is distributed according to $X_{F}^{2}$ with *t* − 1 degrees of freedom. Thus, for any pre-determined level of significance *α*, the null hypothesis will be rejected if $X_{F}^{2}>X_{F}^{\alpha }$. The critical value of $X_{F}^{\alpha }$ for *α* = 0.05 with seven degrees of freedom is 14.07. The Friedman statistic of zero-one loss in Table 4 is 15.32, which is larger than 14.07. Hence, the null-hypotheses is rejected and these classifiers are different.

**Table 4:** The comparison of classification performance between classifiers in terms of zero-one loss, bias, variance and AUC.

	Dataset	NB	AODE^[17]^	TAN^[16]^	KDB^[18]^	AKDB^[11]^	PKDB	LibSVM	Random Forest
**Zero-one loss**	Dis	0.0234 ± 0.0127	0.0241 ± 0.0126	0.0198 ± 0.0101	0.0202 ± 0.0105	0.0193 ± 0.0092	0.0182 ± 0.0087	0.0215 ± 0.0106	0.0185 ± 0.0065
	Hypothyroid	0.0147 ± 0.0095	0.0128 ± 0.0082	0.0138 ± 0.0071	0.0122 ± 0.0062	0.0112 ± 0.0065	0.0093 ± 0.0076	0.0165 ± 0.0054	0.0146 ± 0.0093
	Breast-cancer-w	0.0255 ± 0.0223	0.0383 ± 0.0248	0.0534 ± 0.0204	0.0845 ± 0.0248	0.0531 ± 0.0212	0.0482 ± 0.0105	0.0406 ± 0.0126	0.0395 ± 0.0087
	Haberman	0.2856 ± 0.1052	0.3337 ± 0.0835	0.3812 ± 0.0975	0.3345 ± 0.1025	0.3054 ± 0.0924	0.2656 ± 0.0987	0.2765 ± 0.1541	0.3106 ± 0.0912
	Heart-disease-c	0.1751 ± 0.0694	0.1668 ± 0.0811	0.1876 ± 0.0863	0.2038 ± 0.0933	0.1848 ± 0.0948	0.1732 ± 0.0672	0.2056 ± 0.0974	0.2116 ± 0.0104
	Pima-ind-diabetes	0.2568 ± 0.0745	0.2303 ± 0.0677	0.2850 ± 0.0755	0.3170 ± 0.0586	0.2425 ± 0.0649	0.2178 ± 0.0765	0.2552 ± 0.0835	0.2713 ± 0.0953
**Bias**	Dis	0.0165 ± 0.0092	0.0174 ± 0.0086	0.0193 ± 0.0058	0.0191 ± 0.0062	0.0191 ± 0.0082	0.0181 ± 0.0063	0.0157 ± 0.0078	0.0177 ± 0.0037
	Hypothyroid	0.0116 ± 0.0101	0.0094 ± 0.0092	0.0104 ± 0.0082	0.0096 ± 0.0065	0.0082 ± 0.0046	0.0075 ± 0.0041	0.0115 ± 0.0035	0.0078 ± 0.0014
	Breast-cancer-w	0.0187 ± 0.0091	0.0243 ± 0.0104	0.0143 ± 0.0052	0.0449 ± 0.0102	0.0196 ± 0.0052	0.0201 ± 0.0029	0.0136 ± 0.0058	0.0181 ± 0.0036
	Haberman	0.2332 ± 0.1118	0.2375 ± 0.1091	0.2298 ± 0.0962	0.2301 ± 0.0765	0.2127 ± 0.1053	0.2010 ± 0.1053	0.2236 ± 0.1021	0.2235 ± 0.0924
	Heart-disease-c	0.1368 ± 0.0946	0.1414 ± 0.0652	0.1426 ± 0.0118	0.1697 ± 0.0763	0.1656 ± 0.0932	0.1602 ± 0.0842	0.1563 ± 0.0256	0.1769 ± 0.0245
	Pima-ind-diabetes	0.1957 ± 0.1043	0.1935 ± 0.0973	0.1917 ± 0.0972	0.1944 ± 0.0916	0.1964 ± 0.1096	0.2053 ± 0.0725	0.2015 ± 0.0635	0.2274 ± 0.0626
**Variance**	Dis	0.0069 ± 0.0033	0.0071 ± 0.0027	0.0005 ± 0.0003	0.0011 ± 0.0003	0.0002 ± 0.0001	0.0002 ± 0.0001	0.0082 ± 0.0002	0.0002 ± 0.0001
	Hypothyroid	0.0031 ± 0.0015	0.0034 ± 0.0012	0.0034 ± 0.0009	0.0024 ± 0.0008	0.0025 ± 0.0009	0.0021 ± 0.0003	0.0057 ± 0.0021	0.0087 ± 0.0002
	Breast-cancer-w	0.0014 ± 0.0003	0.0118 ± 0.0081	0.0207 ± 0.0006	0.0504 ± 0.0102	0.0255 ± 0.0093	0.0248 ± 0.0072	0.0213 ± 0.0026	0.0204 ± 0.0026
	Haberman	0.0325 ± 0.0103	0.0312 ± 0.0121	0.0317 ± 0.0093	0.0333 ± 0.0101	0.0322 ± 0.0112	0.0309 ± 0.0097	0.0211 ± 0.0082	0.0324 ± 0.0055
	Heart-disease-c	0.0443 ± 0.0103	0.0463 ± 0.0101	0.0497 ± 0.0092	0.0914 ± 0.0082	0.0744 ± 0.0084	0.0718 ± 0.0045	0.0702 ± 0.0045	0.0823 ± 0.0029
	Pima-ind-diabetes	0.0715 ± 0.0110	0.0729 ± 0.0103	0.0751 ± 0.0097	0.0689 ± 0.0101	0.0661 ± 0.0082	0.0525 ± 0.0064	0.0516 ± 0.0095	0.0689 ± 0.0073
**AUC**	Dis	0.9828 ± 0.2112	0.9773 ± 0.1066	0.9933 ± 0.0924	0.9912 ± 0.1076	0.9905 ± 0.0824	0.9998 ± 0.1142	0.6228 ± 0.1043	0.9666 ± 0.0972
	Hypothyroid	0.7235 ± 0.1237	0.9756 ± 0.2062	0.9807 ± 0.2112	0.9818 ± 0.1637	0.9396 ± 0.1253	0.9887 ± 0.1512	0.7482 ± 0.0624	0.9882 ± 0.1125
	Breast-cancer-w	0.5150 ± 0.0827	0.4572 ± 0.1213	0.7823 ± 0.1046	0.8238 ± 0.1237	0.8480 ± 0.0975	0.9157 ± 0.0882	0.9557 ± 0.1074	0.9877 ± 0.1067
	Haberman	0.7823 ± 0.1162	0.8124 ± 0.0912	0.8637 ± 0.0824	0.8831 ± 0.0967	0.9237 ± 0.1057	0.9742 ± 0.1169	0.5234 ± 0.0824	0.8632 ± 0.0913
	Heart-disease-c	0.5332 ± 0.0474	0.5134 ± 0.0421	0.4528 ± 0.0474	0.4425 ± 0.0518	0.4724 ± 0.0436	0.5153 ± 0.0517	0.8224 ± 0.0613	0.8661 ± 0.1092
	Pima-ind-diabetes	0.6793 ± 0.0626	0.7141 ± 0.0631	0.7367 ± 0.0732	0.7445 ± 0.0974	0.7632 ± 0.0635	0.8124 ± 0.0842	0.7225 ± 0.0623	0.8081 ± 0.1064

Quinlan believed that the two relatively large datasets, i.e. Dis and Hypothyroid, have been corrupted [28] and many missing values exist (6064 missing values in dataset Dis and 5329 missing values in dataset Hypothyroid). When we substitute these missing values with a specific value, i.e., “?” or unknown, noise is artificially introduced and the performance of learned classifier may be degraded. For the other four small datasets with less than 800 instances, the training data provided only accounts for a small portion of the full dataset. Thus the estimation of conditional probability will be of low-confidence. Relatively simple structure resulted from underfitting rather than overfitting may help to improve the classification performance of learning algorithm. From the experimental results of zero-one loss in Table 4 we can see that, classifiers with complex structure don’t necessarily enjoy significant advantage over classifiers with simple structure. For example, KDB, LibSVM and RF perform poorer than NB on datasets Breast-cancer-w and Heart-disease-c. However, ${\text{KDB}}_{P}$ provides an effective way to learn high-confidence dependency relationships implicated in testing instance. RDE can remove the redundant dependency relationships that are irrelevant to class label and add high-confidence conditional dependencies. The negative effect caused by noise and insufficient data will be mitigated to some extent. AKDB performs better than KDB. PKDB even performs the best among all classifiers in terms of zero-one loss.

**Fig 2:**
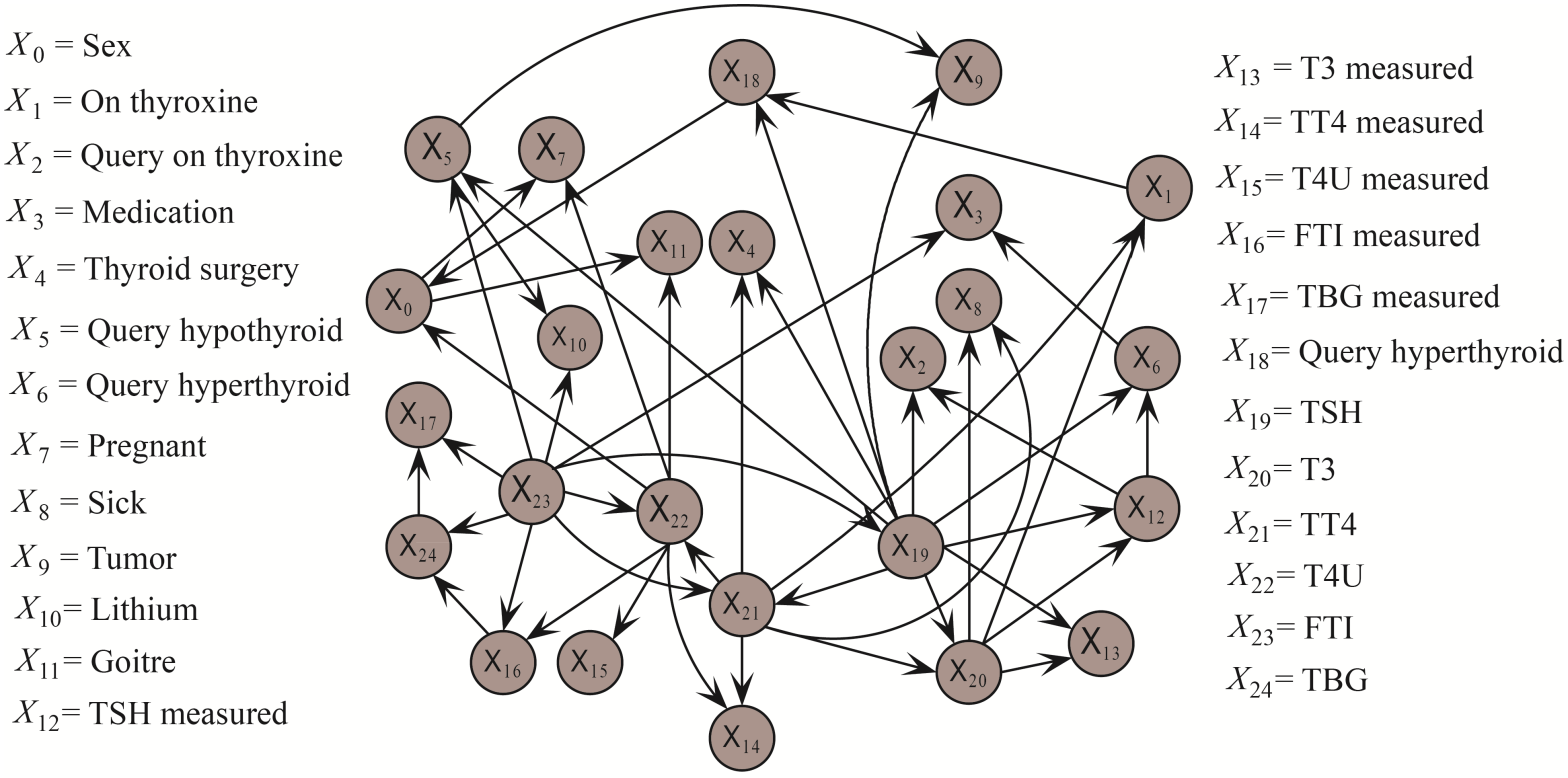
The network structure of KDB(*k* = 2) on dataset Hypothyroid. Class variable *Y* is not included for simplicity. Only conditional dependencies between attributes are shown.

We then clarify from the viewpoint of bias-variance decomposition. The experimental results of variance are reasonable that AODE achieves higher variance than NB because of its complex structure. However, AODE achieves higher bias on dataset Dis, which means underfitting to some extent. Since AODE indiscriminately represents all 29*28 = 812 conditional dependencies, some weak dependencies may represent a large noise component in the training set and counteract the effect the strong dependencies, making it underfit dataset Dis and its prediction less accurate than NB. For dataset Hypothyroid AODE only needs to represent 25*24 = 600 conditional dependencies and negative effect of weak dependencies can be mitigated. When *k* = 2, KDB can represent 0 + 1 + 2⋯ + 2 = 49 conditional dependencies (as shown in Fig 2 whereas TAN only needs to represent 28 conditional dependencies. Thus KDB achieves higher variance since it fits training set well even there exists noise. As a result, the KDB does not fit the testing instance much better than TAN. Noisy training data will reduce the confidence level of the classification model. For classifiers learned from the other four small datasests, overfitting is almost inevitable. KDB, LibSVM and RF perform poorer than NB on datasets Breast-cancer-w and Heart-disease-c in terms of variance. How to reduce variance is a crucial point for improving classification accuracy. RDE helps to mitigate the negative effect of overfitting, thus the variance for PKDB is always lower than that for AKDB and KDB.

AUC is often used to evaluate the classification performance while dealing with imbalanced data. From Table 4 we can see that, TAN and KDB perform better than NB more often than not on small datasets. That indicates although the negative effect caused by overfitting may reduce the classification accuracy, the dependency relationships implicated will help to improve the the discriminatory power of BNCs. The definitions of LMI and CLMI considers all possible values of class variable, thus ${\text{KDB}}_{P}$ cannot overfit the given testing instance P, but provides a possible dependence tree structure to describe the relationships among attribute values in P. The advantage of PKDB over other classifiers in AUC is especially obvious on datasets Dis and Hypothyroid. In contrast, AKDB also uses the personalized ${\text{KDB}}_{P}$, it performs much worse. This can be attributed to the low-confidence dependency relationships mined from these small datasets. LibSVM performs poorer on datasets Dis and Hypothyroid but better on the other four small datasets. RF demonstrates significant robustness while dealing with relatively large or small datasets.

## Discussion

**Fig 3:**
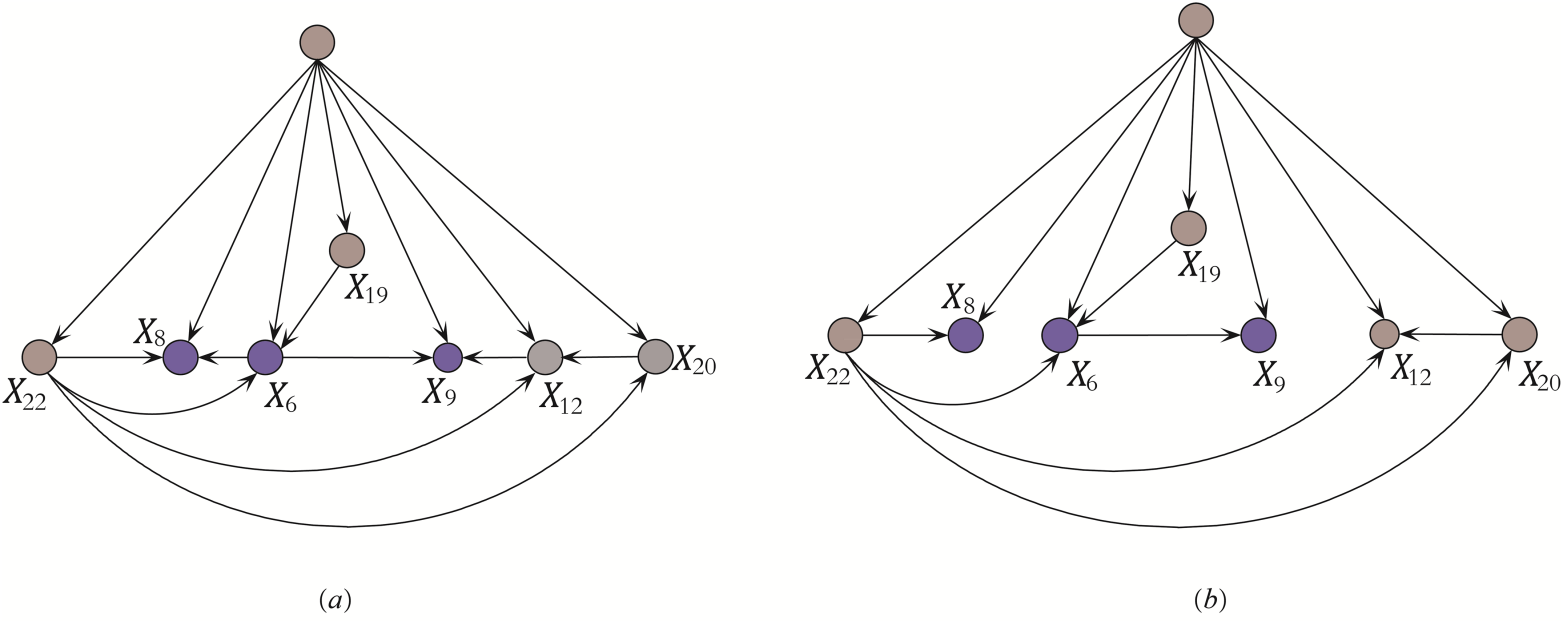
The substructures of ${\text{KDB}}_{P}$ (a) and KDB_*O*_ (b) for {*X*_8_, *X*_6_, *X*_9_} learned from *Case*1 = (*x*_19_ = 43, *x*_23_ = 47, *x*_22_ = 1.26, *x*_20_ = 2, *x*_21_ = 59, *x*_12_ = *y*, *x*_13_ = *y*, *x*_6_ = *t*, *x*_14_ = *y*, *x*_15_ = *y*, *x*_16_ = *y*, *x*_5_ = *f*, *x*_24_ = ?, *x*_17_ = *n*, *x*_1_ = *f*, *x*_0_ = *F*, *x*_18_ = 28, *x*_4_ = *f*, *x*_2_ = *f*, *x*_8_ = *f*, *x*_11_ = *f*, *x*_7_ = *f*, *x*_9_ = *t*, *x*_3_ = *f*, *x*_10_ = *f*). The arcs *X*_6_(Query hyperthyroid) → *X*_8_(Sick), *X*_12_(TSH measured) → *X*_9_(Tumor) in (a) are identified as redundant and removed. As shown in (b), no more attributes with higher ranks are considered as possible parents of *X*_8_ and *X*_9_.

**Fig 4:**
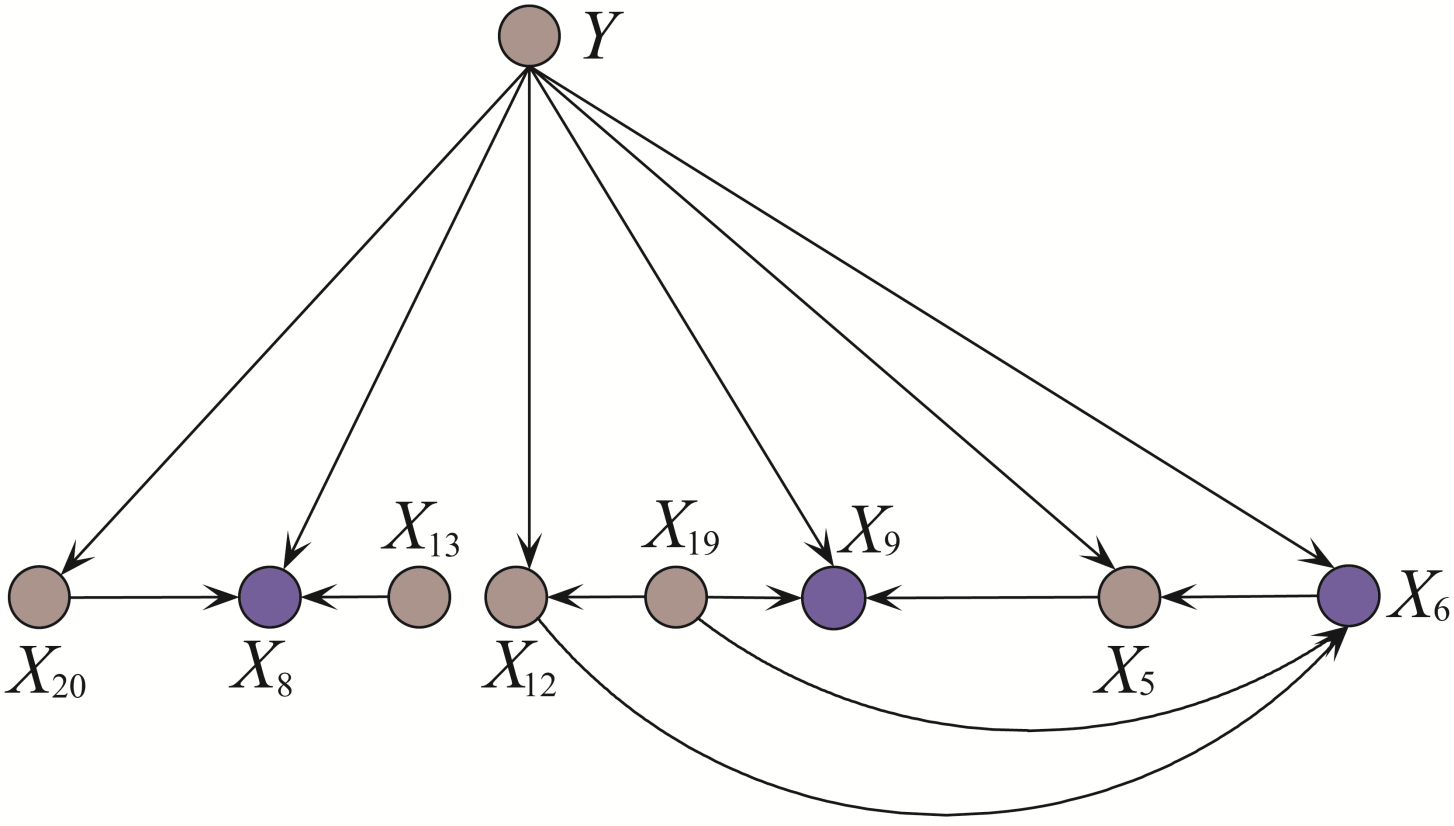
The substructure of KDB for {*X*_8_, *X*_6_, *X*_9_} learned from training set. The arc *X*_5_(TSH measured) → *X*_9_(Tumor) is not reasonable when *X*_9_ = *t* (i.e., ‘true’).

Doctors may need to determine if blood tests are necessary for patients due to their respective risk factors, e.g., family history of goitres, Gender or Age. By computing LMI, CLMI from the local perspective, KDB_*O*_, which learns from individual testing instance, is obviously an example of learners for precision medicine. PKDB can utilize the information provided by the training set and testing instances with the help of the aggregating mechanism. To prove this, we take two cases for example from Hypothyroid dataset, which take different class labels. The first case that is diagnozed as “hypothyroid” is shown as follows

$$\begin{array}{ccc} Case1 & = & (x_{19}=43,x_{23}=47,x_{22}=1.26,x_{20}=2,x_{21}=59,x_{12}=y,x_{13}=y,x_{6}=t,\\ & & x_{14}=y,x_{15}=y,x_{16}=y,x_{5}=f,x_{24}=?,x_{17}=n,x_{1}=f,x_{0}=F,x_{18}=28,\\ & & x_{4}=f,x_{2}=f,x_{8}=f,x_{11}=f,x_{7}=f,x_{9}=t,x_{3}=f,x_{10}=f) \end{array}$$ (15)

where ‘?’ is used to denote a value that is missing or unknown. The attribute values in *case*Â 1 have been sorted by comparing *I*(*x*_*i*_; *Y*). Among them, *x*_19_ or TSH ranks the highest, thus the level of TSH is closely related to some definite results and further tests will be needed. The full network structure with 25 attributes are too complex (47 arcs or conditional dependencies) to explain, so we just select one substructure to clarify. The conditional dependencies in ${\text{KDB}}_{P}$ and KDB, which focus on attributes {*X*_8_, *X*_6_, *X*_9_}, are respectively shown in Figs 3(a) and 4. In Fig 3(a), the testing result of *X*_22_(T4U) can explain why the patient does not feel sick(*X*_8_ = *f*), thus *X*_6_(query on hyperthyroid) does not provide valuable information. The arc *X*_6_ → *X*_8_ is removed. By comparing KDB shown in Fig 4 and KDB_*O*_ shown in Fig 3(b), the limitation of KDB in precise representation is obvious. Hyperthyroidism is a condition in which thyroid gland produces too much of the hormone thyroxine. One symptom for hyperthyroid is an enlarged thyroid gland, which may appear as a swelling at the base of one’s neck. It is reasonable in Fig 3(b) that *X*_9_(tumor) is related to *X*_6_(query on hyperthyroid) whereas in Fig 4 *X*_9_(tumor) is related to *X*_5_(query on hypothyroid). To judge the possibility of hypothyroidism, blood tests (including TSH(*X*_12_), T3(*X*_20_) and T4U(*X*_22_)) are needed. The close relationships can be clearly seen in Fig 3.

The detail of the second instance that is diagnozed as “negative” is shown as follows,

$$\begin{array}{ccc} Case2 & = & (x_{23}=51,x_{21}=37,x_{20}=0.5,x_{19}=9.7,x_{22}=0.72,x_{13}=y,x_{12}=y,x_{1}=t,\\ & & x_{14}=y,x_{15}=y,x_{16}=y,x_{5}=f,x_{0}=F,x_{24}=?,x_{17}=n,x_{4}=f,x_{18}=46,\\ & & x_{6}=f,x_{8}=f,x_{2}=f,x_{11}=f,x_{9}=f,x_{7}=f,x_{3}=f,x_{10}=f) \end{array}$$ (16)

**Fig 5:**
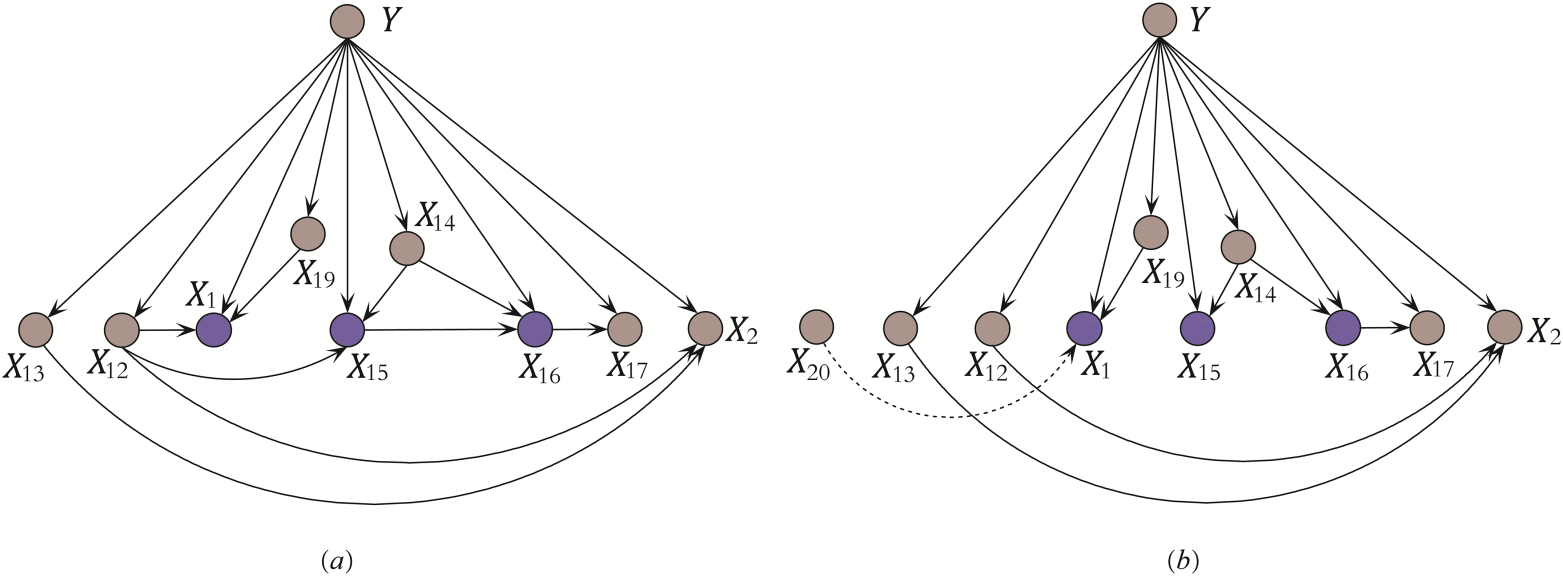
The substructures of ${\text{KDB}}_{P}$ (a) and KDB_*O*_ (b) for {*X*_1_, *X*_15_, *X*_16_} learned from Case2 = (*x*_23_ = 51, *x*_21_ = 37, *x*_20_ = 0.5, *x*_19_ = 9.7, *x*_22_ = 0.72, *x*_13_ = *y*, *x*_12_ = *y*, *x*_1_ = *t*, *x*_14_ = *y*, *x*_15_ = *y*, *x*_16_ = *y*, *x*_5_ = *f*, *x*_0_ = *F*, *x*_24_ = ?, *x*_17_ = *n*, *x*_4_ = *f*, *x*_18_ = 46, *x*_6_ = *f*, *x*_8_ = *f*, *x*_2_ = *f*, *x*_11_ = *f*, *x*_9_ = *f*, *x*_7_ = *f*, *x*_3_ = *f*, *x*_10_ = *f*). The arcs *X*_12_(TSH measured) → *X*_15_(Query hypothyroid) and *X*_15_(T4U measured) → *X*_16_(FTI measured) in (a) are identified as redundant and removed. No more attributes with higher ranks are considered as possible parents of *X*_15_ and *X*_16_. Arc *X*_12_(TSH measured) → *X*_1_(On thyroxine) is substituted with arc *X*_20_(T3) → *X*_1_(On thyroxine).

**Fig 6:**
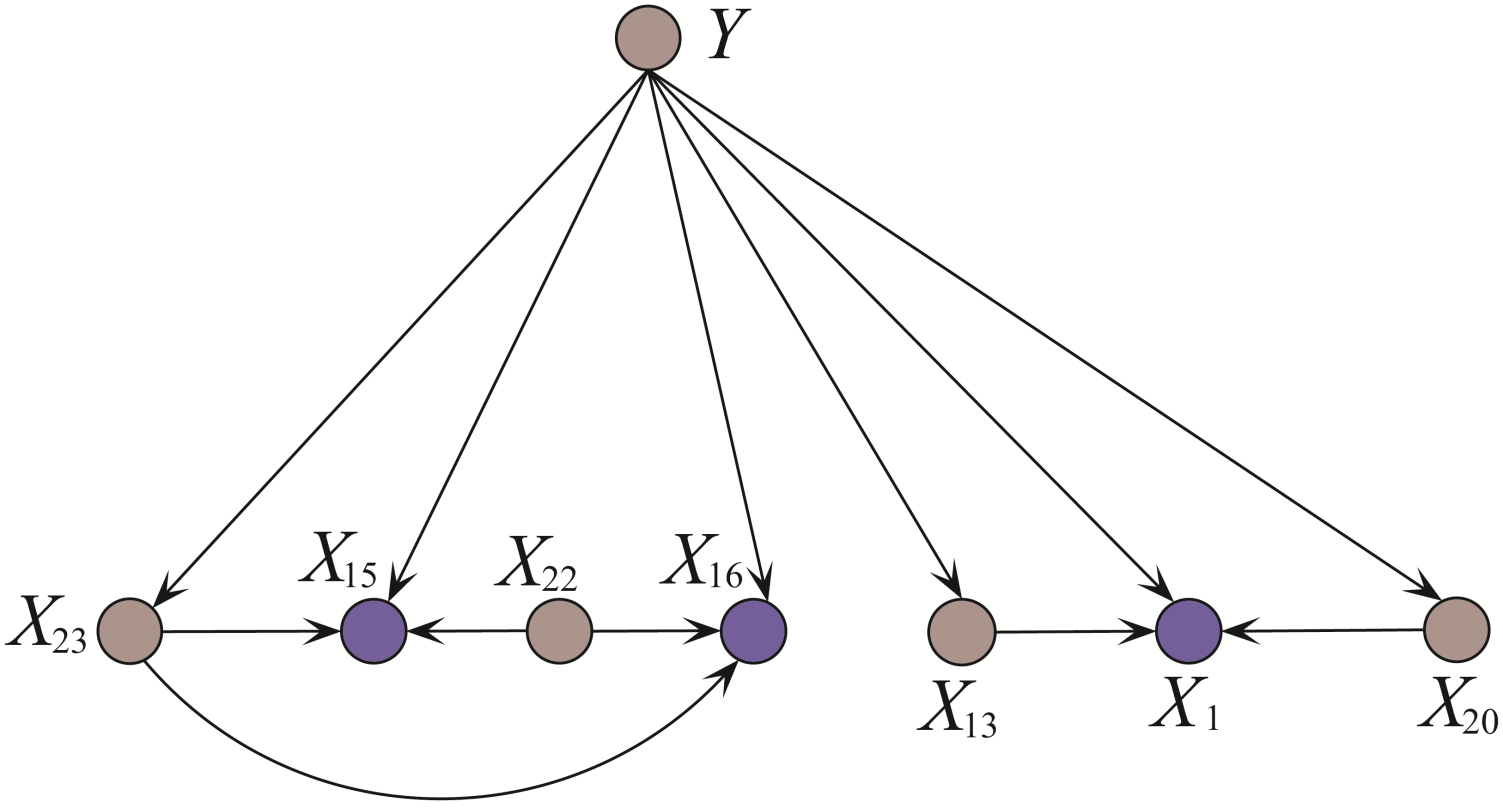
The substructure of KDB for {*X*_1_, *X*_15_, *X*_16_}. The arc *X*_13_ (T3 measured) → *X*_1_ (On thyroxine) is redundant since the information provided by *X*_20_ (T3) includes the information provided by *X*_13_ (T3 measured).

The conditional dependencies in ${\text{KDB}}_{P}$ and KDB, which focus on attributes {*X*_1_, *X*_15_, *X*_16_}, are respectively shown in Figs 5(a) and 6. The information implicated in some attribute values may overlap or even cover that in other attribute values. For example, “TSH measured = *y*” is a premise of “TSH = 4.6”. “Sex = *F*” is a premise of “Pregnant = *t*”. Although there exist strong dependencies between these attribute values and they may appear simultaneously as the co-parents of some attributes, this kind of dependencies are redundant and should be substituted. The arc *X*_12_ → *X*_1_ is removed from Fig 5(a) and we should find another parent for *X*_1_ as shown in Fig 5(b). To provide accurate diagnosis for hypothyroid, the blood tests of TT4 and FTI are always used simultaneously. Thus the arc *X*_15_ → *X*_16_ is also redundant and should be removed. The limitation of KDB in scalability is obvious. As shown in Fig 6, the value of *X*_13_(T3 measured) is a premise of the value of *X*_20_(T3). When they appear as the co-parents of some other attribute, e.g., *X*_1_, the conditional probability *P*(*x*_1_|*x*_13_, *x*_20_, *y*) will approximate the estimate of *P*(*x*_1_|*x*_20_, *y*). *X*_13_(T3 measured) cannot provide any valuable information to *X*_1_.

## Conclusion and future work

${\text{KDB}}_{P}$ takes instance P as the target and its network structure describes the dependency relationships in P. Because of the computational overhead, only a limited number of dependencies, which are determined by parameter *k*, can be described by ${\text{KDB}}_{P}$. The proposed approach, RDE, is a filter that transforms the testing instance to substitute these redundant dependencies with other dependencies at classification time. The experimental results show that the classification accuracy (or zero-one loss) and robustness (bias and variance) are significantly enhanced by the addition of RDE. Besides, the dependency relationships that RDE identified in testing instance are irrelevant to class label, thus it is especially applicable to imbalanced data, e.g. Dis and Hypothyroid. That may be the main reason why RDE obtains the highest AUC values among all the BNCs on the datasets Dis and Hypothyroid.

RDE searches for the mapping relationships between specific attribute values and then identifies redundant ones. Thus it is suited to probabilistic techniques which deal with discrete attributes, such as KDB. RDE can also be extended to deal with continuous attributes. One possible solution is that, if the conditional probability density function *p*(*x*_*j*_|*x*_*i*_) is relatively high (or greater than a specified value *δ*) then the mapping relationship *x*_*i*_ → *x*_*j*_ is supposed to exist and *x*_*j*_ is redundant. The estimation of *p*(*x*_*j*_|*x*_*i*_) should be learned reliably from training data and the data size should be very large. Although the estimation of *p*(*x*_*j*_|*x*_*i*_) will be time-consuming and more experimental study is needed to determine the value of *δ* for different attributes, the research work on extending RDE is still very promising.
